# Nanocarrier systems loaded with IR780, iron oxide nanoparticles and chlorambucil for cancer theragnostics

**DOI:** 10.3762/bjnano.15.17

**Published:** 2024-02-06

**Authors:** Phuong-Thao Dang-Luong, Hong-Phuc Nguyen, Loc Le-Tuan, Xuan-Thang Cao, Vy Tran-Anh, Hieu Vu Quang

**Affiliations:** 1 NTT Hi-tech institute, Nguyen Tat Thanh University, Ho Chi Minh City, 700000, Vietnam; 2 Faculty of Chemical Engineering, Industrial University of Ho Chi Minh City, Ho Chi Minh City, 700000, Vietnam; 3 Institute of Applied Technology and Sustainable Development, Nguyen Tat Thanh University, Ho Chi Minh City 700000, Vietnam; 4 Department of Biotechnology, NTT Hi-tech institute, Nguyen Tat Thanh University, Ho Chi Minh City, 700000, Vietnam

**Keywords:** cancer, chlorambucil, F127-folate, IR780, iron oxide nanoparticles, PLGA, theragnostics

## Abstract

Theragnostics has become a popular term nowadays, since it enables both diagnosis and therapy at the same time while only using one carrier platform. Therefore, formulating a nanocarrier system that could serve as theragnostic agent by using simple techniques would be an advantage during production. In this project, we aimed to develop a nanocarrier that can be loaded with the chemotherapeutic medication chlorambucil and magnetic resonance imaging agents (e.g., iron oxide nanoparticles and near-infrared fluorophore IR780) for theragnostics. Poly(lactic-*co*-glycolic acid) was combined with the aforementioned ingredients to generate poly(vinyl alcohol)-based nanoparticles (NPs) using the single emulsion technique. Then the NPs were coated with F127 and F127-folate by simple incubation for five days. The nanoparticles have the hydrodynamic size of approx. 250 nm with negative charge. Similar to chlorambucil and IR780, iron oxide loadings were observed for all three kinds of NPs. The release of chlorambucil was quicker at pH 5.4 than at pH 7.4 at 37 °C. The F127@NPs and F127-folate@NPs demonstrated much greater cell uptake and toxicity up to 72 h after incubation. Our in vitro results of F127@NPs and F127-folate@NPs have demonstrated the ability of these systems to serve as medication and imaging agent carriers for cancer treatment and diagnostics, respectively.

## Introduction

Theragnostic nanoparticles (NPs) are a diagnostic and therapeutic delivery system. The delivery system is comprised of three components: the carrier, the imaging agent, and the therapeutic drug, all of which need clinical approval before being used in humans.

Poly(lactic-*co*-glycolic acid) (PLGA) is an approved biodegradable and biocompatible material for clinical use [1]. Particularly, PLGA is widely used for nanoparticle formulation because it is a versatile material that can serve multiple functions, including transporting of therapeutic drugs and imaging agents [2–3]. In addition, PLGA can be altered in numerous ways to increase the half-life of nanoparticles in the blood and target efficacy. To increase the blood half-life, stealth materials have been attached to the nanoparticle surface to prevent protein adsorption and immune cell phagocytosis [4]. Most sheath materials are hydrophilic polymers such as poly(vinyl alcohol), poly(ethylene glycol), and poly(ethylene oxide) (PEO). To improve the targeting ability of nanoparticles, ligands are typically designed to be located on the exterior of nanoparticles. Typically, ligands are cell-type-specific monoclonal antibodies, RGD peptides for the overexpression of the asialoglycoprotein receptor on cancer cells [5], mannose for the mannose receptor on activated macrophages [6–7], and folic acid for the overexpression of the folate receptor on the surface of cancer cells and activated macrophages [8]. Thus, in this study, PLGA was chosen for NP formulation since it is a biocompatible and biodegradable material.

Clinical use of superparamagnetic oxide nanoparticles (SPIONs) has been authorized [9]. SPIONs have been utilized in magnetic particle imaging (MPI), magnetic resonance imaging (MRI), computer tomography (CT), and additional imaging models [9–11]. SPIONs have been modified to be applicable to a variety of fields. However, SPIONs typically serve as the core of nanoparticles, while the outer shell is composed of stabilization coating materials such as chitosan, mannan, poly(ethylene glycol) (PEG), Pluronic™ F-127, or PLGA [7,12–13]. SPIONs stabilized with PLGA have attracted interest due to their high potential applications in various fields, including theragnostics. The PLGA SPION nanoparticles were modified to carry siRNA for silencing the inflammatory cytokine Cox-2 in activated macrophages and to serve as a tracer for locating activated macrophages in a mouse model of intra-uterine urinary obstruction [14]. In another study, PLGA SPIONs could transport chemotherapeutic agents for cancer treatment and diagnosis [15]. Therefore, the combination of PLGA and SPIONs promises a useful theragnostic system in our study.

In addition to the encapsulation of SPIONs in PLGA employed for diagnostics, near-infrared (NIR) fluorescent dyes are a potential alternative for the diagnosis and excision of residual malignant cancer, which is invisible by conventional visual examination and palpation [16–18]. Indocyanine green is the only NIR dye permitted for clinical use [19]. Dyes such as IR780 and IR783 are also promising diagnostic choices. Encapsulation of IR780 in nanoparticles can be used for imaging and photothermal, photodynamic, and combinatorial cancer therapies [20–22]. IR780 is also utilized in PEG-PLA nanoparticles for photodynamic therapy of human breast cancer cells [23–24]. Thus, using IR780 in our NPs would bring advantages for local imaging and treatment.

Chemotherapy medications assist in inhibiting the development of tumors. Chemotherapeutic agents such as doxorubicin attach to chromosomes and inhibit DNA replication, while paclitaxel depolymerizes the cytoskeleton and chlorambucil (CHL) inhibits DNA synthesis. These drugs can be encapsulated inside nanoparticles for administration to increase the stability of the medication in circulation and therapeutic efficacy. For example, doxorubicin can be inserted into liposomes and paclitaxel attaches to the protein particle [25–26]. PLGA is one of the finest materials for transporting chemotherapy drugs. PLGA transports not only hydrophobic but also hydrophilic drugs. The encapsulation of chemotherapeutics in PLGA nanoparticles has been extensively studied. PLGA has been loaded with doxorubicin, for instance, for tumor treatment [27], and PLGA-chlorambucil nanoparticles have been developed for the treatment of breast cancer [28]. Due to the efficiency of CHL in cancer treatment, CHL has been used as a drug model in order to evaluate our formulated NPs.

Therefore, in this study, we propose to develop a carrier system capable of transporting NPs that could carry iron oxide (IO) nanoparticles, IR780, and CHL for cancer theragnostic applications.

## Materials and Methods

Chlorambucil (C0253), PLGA 504H (719900), IR780 (425311), FeSO_4_·7H_2_O (215422), FeCl_3_·6H_2_O (236489), NaOH (221465), oleic acid (364525), Pluronic™ F-127 (P2443), poly(vinyl alcohol) (PVA) (P8136), phosphate buffered saline (PBS) (P4417), dimethyl sulfoxide (DMSO) (472301), dichloromethane (DCM), 3-[4,5-dimethylthiazol-2-yl]-2,5 diphenyl-2*H*-tetrazolium bromide (MTT) (M2128), and coumarin-6 (442631), K_3_[Fe(CN)_6_] (1049730100) were purchased from Sigma-Aldrich. Dulbecco's Modified Eagle Medium (DMEM) (11965092), fetal bovine serum (FBS) (MT35010CV), antibiotic (15-240-062), and trypsin (25-200-056) were purchased from Gibco, Fisher Scientific. All other solvents and reagents were of chemical grade.

### Synthesis of iron oxide nanoparticles

Iron oxide NPs were synthesized using a modified coprecipitation method [13]. Firstly, 10 mmol of FeSO_4_·7H_2_O and 5 mmol of FeCl_3_·6H_2_O were dissolved in 50 mL of deionized water in a N_2_ atmosphere. Then, 1 M of NaOH was carefully added into the solution while swirling until the pH reached 14. Next, 1 mL of oleic acid was added to the solution and heated to 70 °C for 30 min. The synthesized IO NPs were washed three times with 50 mL of DCM and stored at −80 °C for further experiments.

### Synthesis of folate-F127

The synthesis was done as previously described [13]. The Fourier-transform infrared spectroscopy (FTIR) results were displayed in the Supporting Information File 1 (Supplementary data 1) and Figure S1.

### Formulation of nanoparticles F127-folate@PLGA/IO/CHL/IR780 and others

In 2 mL of DCM, 20 mg of PLGA, 40 μg of IO, 2 mg of CHL, and 0.1 mg of IR780 were mixed. The organic solvent was then added to the aqueous phase, which contained 10 mL of 1.5% PVA. The mixture was then emulsified by vortexing at 1000 rpm for 1 min, followed by sonication (Sonics, Vnibra cells, USA) over an ice bath for 1 min at 40 W, 40%, 10 s pulse, and 2 s rest. Then, the organic phase was magnetically stirred at 200 rpm and allowed to evaporate overnight in a dark area at room temperature. In the next day, the nanoparticles were centrifuged at 12,000 rpm for 30 min, then resuspended in 600 µL of distilled water. The nanoparticles were then separated into three 200 μL suspension tubes. Then, 2 mg of F127-folate or 2 mg of F127 was separately added to the tubes, and the ligand exchange was performed at 4 °C for one week. The tube without added polymer was designated as PVA@PLGA/IO/CHL/IR780 (PVA@NP). Then, the nanoparticles were centrifuged for 30 min at 12,000 rpm and resuspended in distilled water. The final formulations of the three nanoparticles were F127-folate@PLGA/IO/CHL/IR780 (F127-folate@NP), F127@PLGA/IO/CHL/IR780 (F127@NP), and PVA@PLGA/IO/CHL/IR780 (PVA@NP).

Coumarin-6 (0.2 mg) was added to the organic phase to create F127-folate@NP/Cou-6, F127@NP/Cou-6, and PVA@NP/Cou-6 for the fluorescence assay in cells.

### Hydrodynamic size and zeta potential

Dynamic light scattering (DLS) and zeta potential spectra were obtained for three replicates on a nanoPartical Horiba SZ-100 (Japan) with the scattering angle of 90° to determine the size distribution and stability of the nanoparticles. The DLS measurements were performed in both 0.1× PBS (13.7 mM of NaCl, 0.27 mM of KCl, 1 mM of Na_2_HPO_4_, and 0.18 mM of KH_2_PO_4_, pH 7.4) and animal cell culture media containing DMEM and 10% FBS.

### Scanning electron microscopy

For scanning electron microscopy (SEM) experiments, 10 μL of F127-folate@NP was loaded on the silica film for 1 min, and water was allowed to evaporate. Then, the NPs were coated with titanium and SEM images were acquired using a FE-SEM S4800 HITACHI, Japan.

### Loading capacity

Entrapment and release of CHL and IR780 in the NPs were measured by the absorbance of CHL at 256 nm and IR780 at 780 nm using HPLC 1200 and NanoDrop One^C^ (Thermoscientific, USA).

In brief, a small amount of freeze-dried NPs was weighted and dissolved in 20 μL of acetonitrile. Then, 80 μL of methanol was added to extract the drug. The mixture was centrifuged at 12,000 rpm for 30 min, and the supernatant was kept for drug entrapment calculation.

### Chlorambucil release

The NPs were kept in 0.1× PBS at pH 7.4 and pH 5.4 for 24, 48, 72, and 168 h at 37 °C. The NPs were then centrifuged at 12,000 rpm for 30 min. The pellet was collected and freeze-dried. The remaining CHL concentration in NPs was calculated as previously described.

### Determination of iron content with the Prussian blue protocol

The iron content was determined by measuring absorption of Prussian blue at 562 nm (Biotek ELX800, Agilent, USA). Briefly, the material was initially reduced for 10 min in 4% HCl. Then, 4% K_3_[Fe(CN)_6_] was added and the mixture was incubated for an additional 10 min.

### Cell culture

The cell lines HepG2, MCF-7, 3T3, and Hek were cultured in DMEM, 10% FBS, and 1% antibiotic at 37 °C and 5% CO_2_.

### Cell uptake estimation

The cells were seeded onto 6-well plates at 100,000 cells/well and were cultured overnight. On the next day, the cells were incubated with 0.5 mg of F127-folate@NP/Cou-6, F127@NP/Cou-6, and PVA@NP/Cou-6 in 1 mL of cell culture media for 4 h. The cells were then washed with PBS three times and trypsinized. The harvested cells were counted and 20,000 cells/well were added to a black 96-well plate in triplicates. The culture medium and cells without treatment were used as controls. Fluorescent signals were obtained at an excitation wavelength of 460 nm and emission wavelength of 510 nm (Victor Nivo, Perkin Elmer, USA). After the measurements, the cell signal was subtracted from the signal from the cells that did not receive treatment. Then, the signals were normalized to the signal of PVA@NP/Cou-6 for comparison.

The fluorescence image of the NPs in the cells were displayed in Supporting Information File 1, Supplementary data 2 and Figure S2.

### Cytotoxicity effects of nanoparticles

The cells were seeded onto 96-well plates at 5,000 cells/well one day prior to the tests. Then, the cells were treated with various particle concentrations (0.5 mg/ mL, 1 mg/mL, and 1.5 mg/mL). Cells treated with CHL and untreated cells were used as controls. Cells were incubated with NPs for 48 and 72 h. The cell viability was evaluated by the MTT assay, and the absorbance was read at 562 nm (Biotek ELX800, Agilent, USA).

## Results

### The morphology, size, and zeta potential of the particles

**Figure 1:**
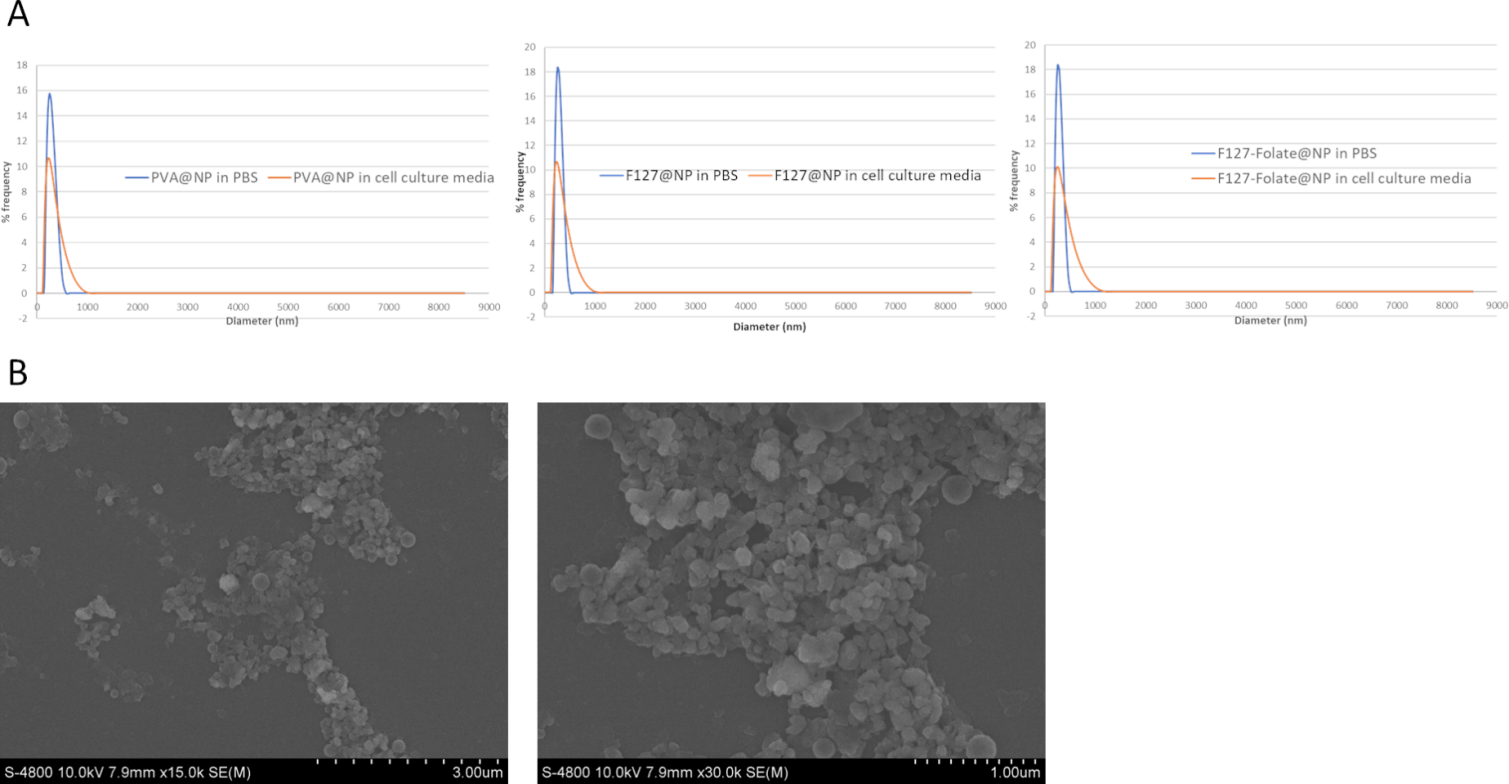
(A) DLS results for the NPs. (B) SEM images of F127-folate@NP at 15k× and 30k× magnifications.

The hydrodynamic size of the three types of NPs (Figure 1A) ranged from 245 ± 11 nm to 246 ± 2 nm with the polydispersity index (PDI) smaller than 0.12, which shows the similarity in size and highly homogeneity among the NPs (Table 1). The zeta potential values of the nanoparticles were: PVA@NP: −46 ± 0.7 mV; F127-folate@NP: −67.4 ± 2.3 mV, and F127@NP: −81.13 ± 2.4 mV (Table 1). It was also showed that when the NPs were in cell culture media, their values of DLS and PDI increased to approx. 280 nm and 0.24, respectively. The NPs were still well dispersed in cell culture media; however, the size increase suggested the adsorption of FBS proteins onto the NPs.

**Table 1:** Hydrodynamic size and polydispersity index of the NPs in PBS and cell culture medium.

	DLS (nm)	PDI	Zeta potential (mV)

F127@NP in 0.1× PBS	256 ± 11	0.12 ± 0.03	−67.4 ± 2.3
F127-folate@NP in 0.1× PBS	245 ± 2	0.09 ± 0.06	−81.13 ± 2.4
PVA@NP in 0.1× PBS	246 ± 1	0.115 ± 0.03	−46 ± 0.7
F127@NP in cell culture media	290 ± 19	0.464 ± 0.07	–
F127-folate@NP in cell culture media	285 ± 18	0.459 ± 0.06	–
PVA@NP in cell culture media	275 ± 14	0.476 ± 0.04	–

The SEM results (Figure 1B) showed the actual size of the nanoparticles, which had a round shape of approx. 150 nm. However, there was a polymer layer covering the outside of the NPs, which made the spherical shape of the NPs not clear.

### Drug loading and release

#### Entrapment of chlorambucil

The entrapment of chlorambucil in the NPs was calculated by measuring the OD absorption of CHL in NPs after purification and freeze-drying. The results showed that there was approx. 0.5% of CHL in the NPs (Table 2). The encapsulation efficiency of the single emulsion was approx. 5%. However, it was reported that the encapsulation efficiency could be increased up to 92% using the double emulsion method [28] or to 70% using the nanoprecipitation method [29].

**Table 2:** The entrapment of chlorambucil, IR780, and iron oxide nanoparticles into NPs.

	Drug loading capacity

chlorambucil	0.5%
IR780	0.9%
iron oxide nanoparticles	1.11%

#### The loading of iron oxide nanoparticles

The entrapment of iron oxide NPs into PLGA nanoparticles was estimated by measuring the absorbance at 562 nm through the Prussian blue reaction. The entrapment of IO was approx. 1.11% of the total weight of nanoparticles (Table 2).

#### The loading of IR780 into nanoparticles

The IR780 loading capacity of the nanoparticles was evaluated by measuring the absorbance at 780 nm. Approximately 0.9% of IR780 was composed of PLGA nanoparticles (Table 2).

#### The release of chlorambucil from the nanoparticles

**Figure 2:**
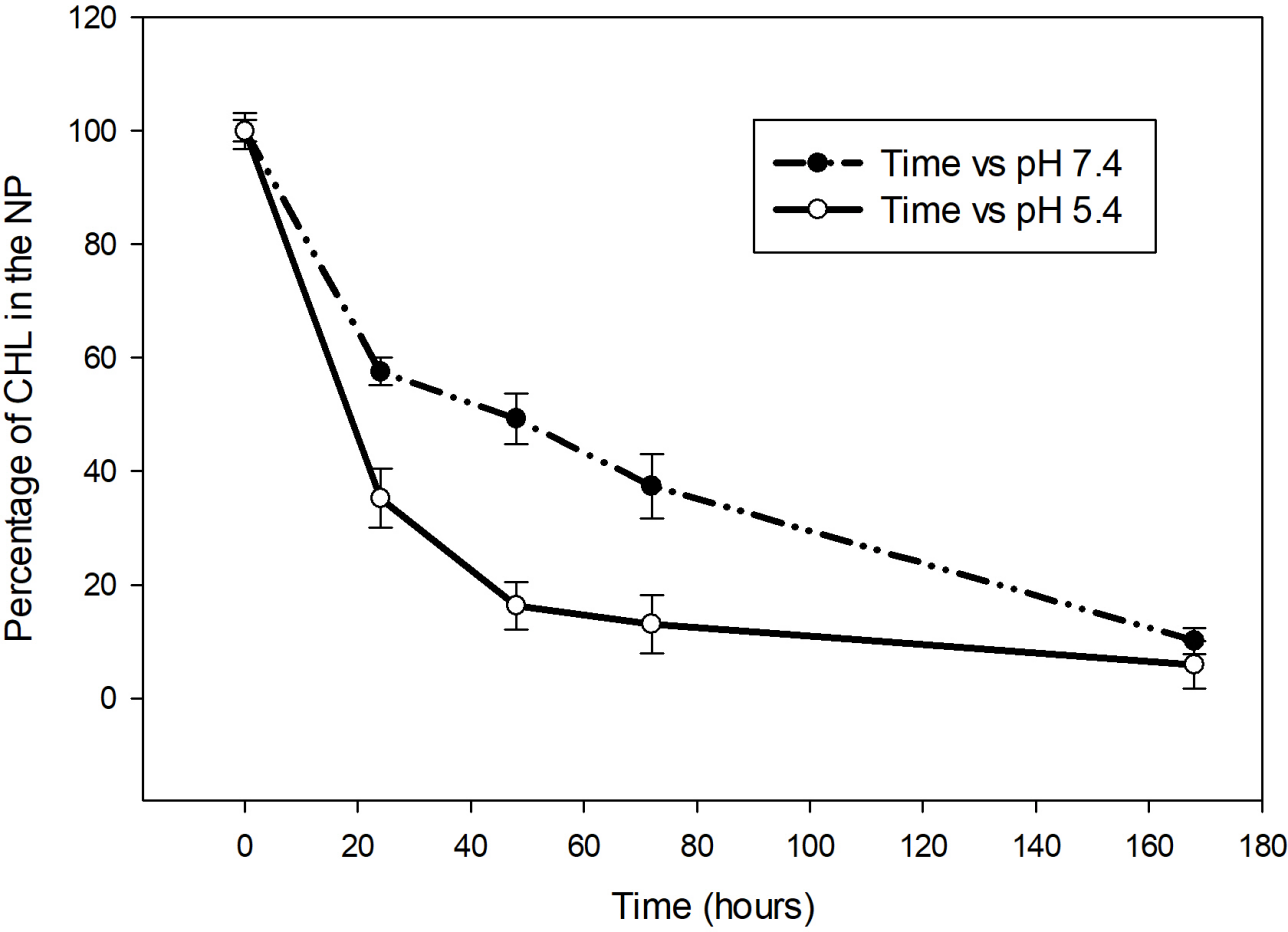
The release of chlorambucil from F127@NP at pH 7.4 and pH 5.4 after seven days of incubation at 37 °C. *n* = 3.

The release of chlorambucil from the NPs was monitored during seven days of incubation in media with pH values of 7.4 and 5.4 at 37 °C, which mimicked physiological and endosomal conditions (Figure 2). After seven days of incubation, the CHL concentration in nanoparticles was maintained between 6% and 10%. The CHL remained in the NPs at 57.6 ± 2.4% and 35.26 ± 5.2% during incubation at pH 7.4 and pH 5.4, respectively, over the first 24 h. After 48 h, the concentration decreased to 49.2 ± 4.5% at pH 7.4 and 16.3 ± 4.2 at pH 5.4. On day three, the CHL concentration in the NPs was 32.9 ± 5.7% at pH 7.4 and 13 ± 5.1% at pH 5.4. After 24, 48, and 72 h, the CHL levels in the NPs were significantly lower when incubated in pH 5.4 medium compared to that in pH 7.4 medium. The faster CHL drug release at pH 5.4 was due to a faster degradation of PLGA at pH 5.4 than that at pH 7.4 [30].

### Targeting of nanoparticles to the cells

**Figure 3:**
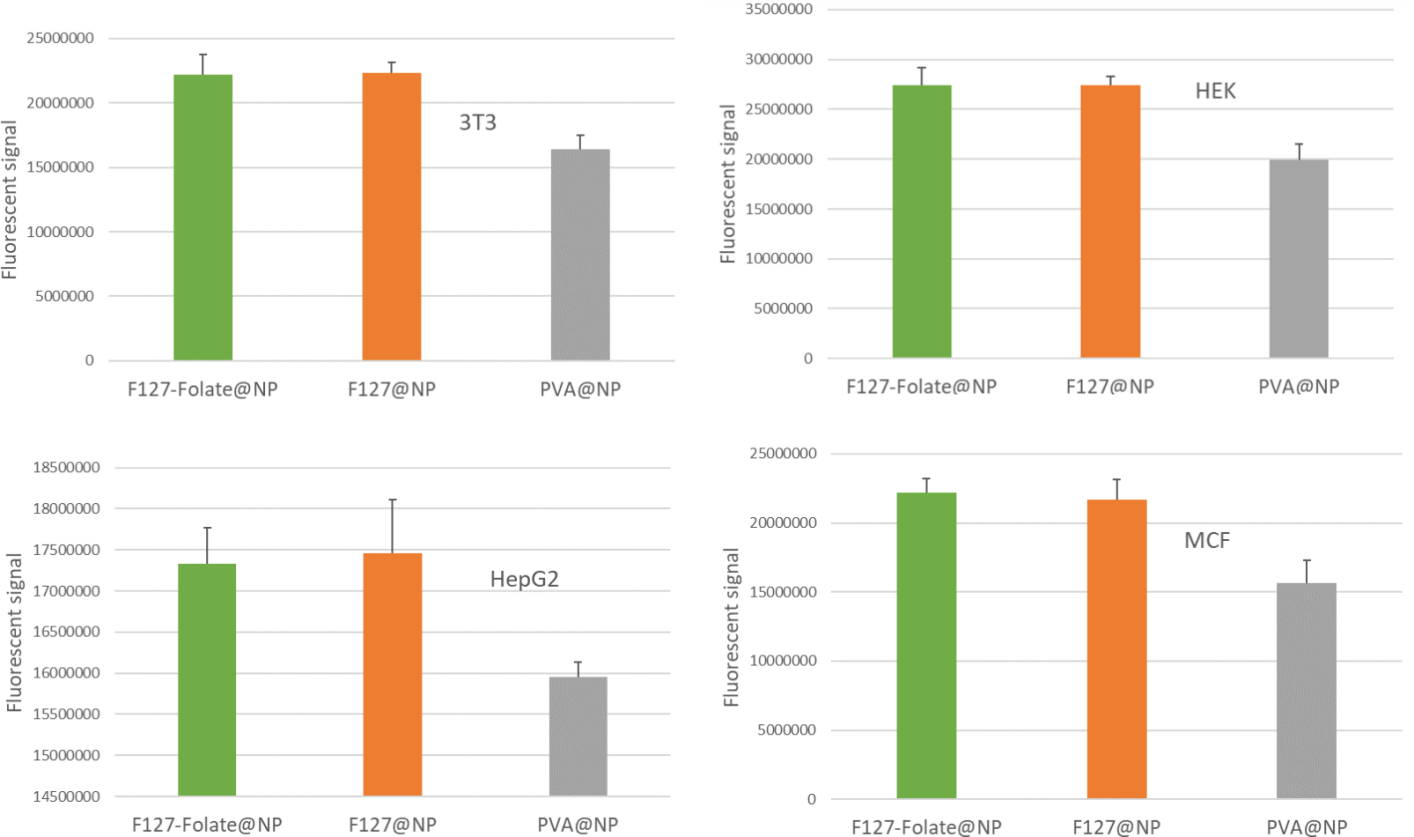
The fluorescence signal of F127-folate@NP/Cou-6, F127-folate@NP/Cou-6, and PVA@NP/Cou-6 in 3T3, HEK, HEPG2, and MCF-7 after background subtraction (*n* = 3).

The NPs encapsulated with Cou-6 were utilized to demonstrate the capacity of our NPs to target cells. In a 6-well plate, cells were treated with 0.2 mg/mL of F127-folate@NP/Cou-6, F127-folate@NP/Cou-6, and PVA@NP/Cou-6 for 3 h at 37 °C and 5% CO_2_. Cells treated with F127-folate@NP/Cou-6 and F127-folate@NP/Cou-6 had stronger fluorescence signals than cells treated with PVA@NP/Cou-6 (Figure 3). This was true for all four types of cells. In 3T3, HEK, and MCF-7 cells, the signal of F127-folate@NP/Cou-6 and F127@NP/Cou-6 was 1.4 times stronger than that of PVA@NP/Cou-6. However, in HepG2 cells, it was only 1.2 times stronger. There was no significant difference between the fluorescence signals of F127-folate@NP/Cou-6 and F127@NP/Cou-6 in any cell type. The appearance of F127-folate@NP/Cou-6 and F127@NP/Cou-6 to MCF7 and HepG2 was also confirmed (Supporting Information File 1, Figure S2). The F127-folate@NP did not show a superior targeting effect to cancer cells, which might be due to the fact that cancer cells were cultured in normal cell culture media. Therefore, the expression of folate receptor was not as high compared to that of cancer cells that grew in folic-acid-free cell culture media.

### Cytotoxicity of the nanoparticles

**Figure 4:**
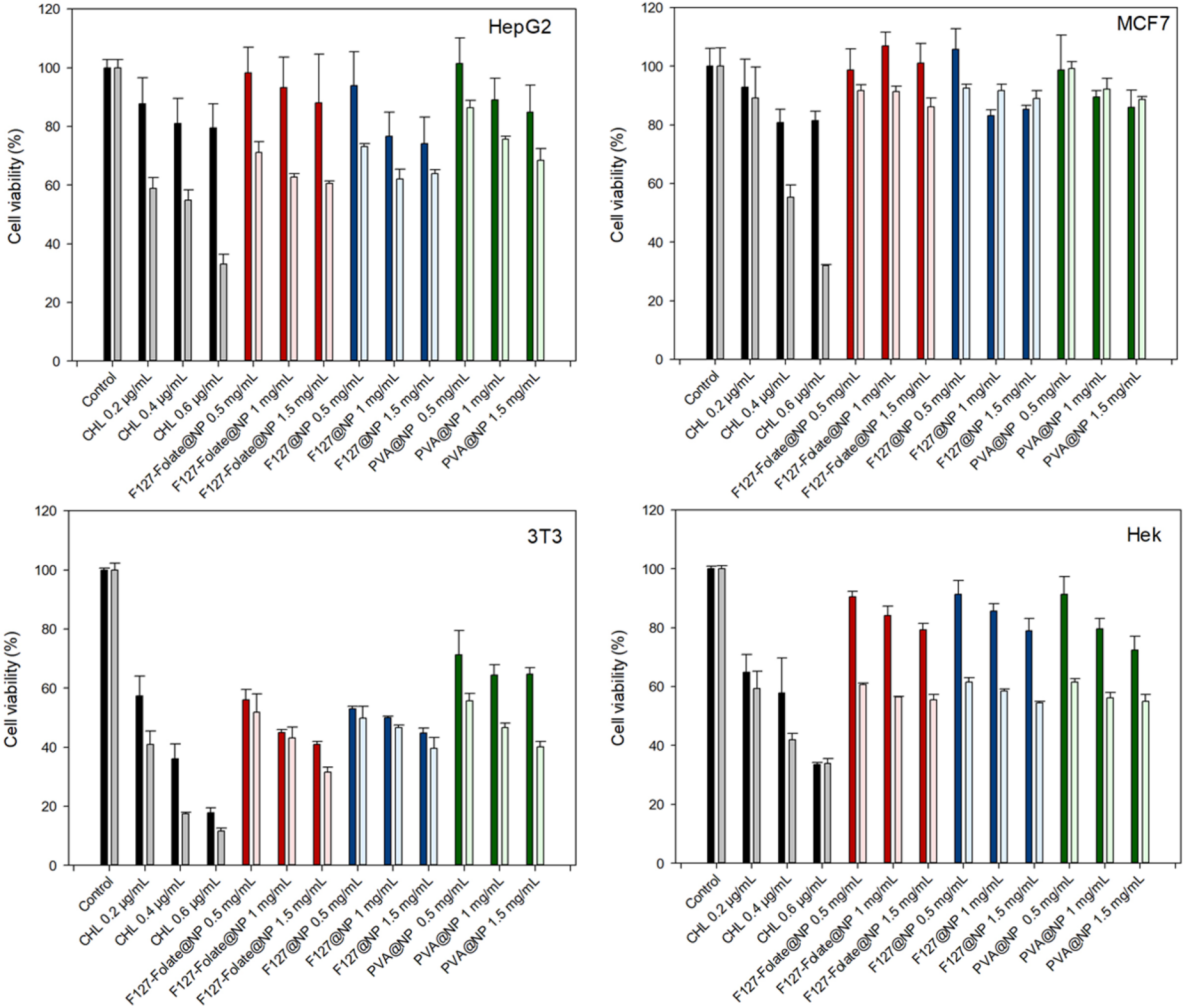
MTT assays of the nanoparticles incubated with HepG2, MCF7, 3T3, and HEK for 48 h (dark color) and 72 h (light color). Cells (5,000 cells/well) were treated with CHL 0.2 μg/mL, 0.4 μg/mL and 0.6 μg/mL (black), F127-folate@NP (red), F127@NP (blue), and PVA@NP (green) at 0.5 mg/mL, 1 mg/mL, 1.5 mg/mL.

This experiment was conducted to assess the toxicity of CHL nanoparticles to four distinct cell types (Figure 4).

After 72 h of incubation, the IC50 of CHL for HepG2 was 0.45 µg/mL. After 72 h of NP exposure to HepG2, the cell viability at the highest dose (1.50 μg/mL) was reduced to approximately 60%. At 72 h, the viability was lower than that at 48 hours. The cellular toxicity at concentrations of 1 mg/mL and 1.5 mg/mL at 48 and 72 h was the same. At 72 h, the concentration of F127@NP and F127-folate@NP was significantly lower than that of PVA-NP. No significant differences existed between F127@NP and F127-folate@NP.

The IC50 of CHL for MCF-7 was 0.4 μg/mL. After 72 h of incubation, the NPs had some impact on the MCF-7 cells. At a dosage of 1.5 mg/mL, the cell viability of both F127@NP and F127-folate@NP decreased to approximately 50%. These results were below PVA@NP (75%). Similar cell viability results were observed with F127-folate@NP and F127@NP.

Chlorambucil was very toxic to 3T3 cells, with an IC50 of 0.4 µg/mL at 48 h and 0.35 mg/mL at 72 h. At the lowest nanoparticle concentration (0.5 mg/mL), the cell viability was approx. 50% for all NPs tested. At a concentration of 0.5 mg/mL, the concentrations of F127 and F127-folate@NP were approx. 50% after 48 and 72 h, whereas the concentrations of PVA@NP were 71% and 55.6% after 48 and 72 h, respectively. The cell viability decreased when the concentration of used NP increased.

In the HepG2 cell line, the IC50 of CHL was 0.45 μg/mL at 72 h. However, cell viability even at the highest concentration of NPs (1.5 mg/mL) was only about 60%. The cytotoxicity of the NPs was significantly lower after 72 h of incubation than that at 48 h. The toxicity effect was similar for both NP concentrations of 1.0 and 1.5 mg/mL at both 48 and 72 h. The cytotoxicity results for F127@NP and F127-folate@NP was significantly lower at 72 h than that for PVA@NP. There were no statistically significant differences between F127@NP and F127-folate@NP.

## Discussion

Nanomedicines have their applications in a number of cancer treatments and diagnoses, including tumor-targeted drug delivery, hyperthermia, photodynamic therapy, and imaging. Nanomedicines can be made from a variety of inert, biodegradable, and in vivo biocompatible materials. Poly(lactic-*co*-glycolic acid) is one of the most biodegradable and biocompatible copolymers owing to its nontoxic breakdown products [3]. It has been encapsulated with a variety of drugs. One of the contemporary concerns is theragnostics, in which PLGA nanoparticles have dual diagnostic and therapeutic roles. Various imaging agents and medications have been encapsulated in PLGA for this purpose. For theragnostic applications, we have produced PLGA nanoparticles that carry chlorambucil as the chemotherapeutic medication and iron oxide nanoparticles as the imaging agent.

Li et al., 2023, published similar results on F127@PLGA nanoparticles and claimed that the F127 polymer flocculates over the PVA layer when incubated with PVA@PLGA nanoparticles [31]. Their research yielded similar findings to our own results. First, F127 could briefly switch the ligand between PVA and F127, or F127 PPO block could stick to the hydrophobic outer shell of PLGA nanoparticles. The presence of F127 on PLGA@NP decreases their charge (Table 1). The alteration of NPs following the hardening of PLGA NPs apparently had no effect on the loading of drugs (CHL) and imaging agents (IR780 and IO) into NPs (Table 2).

Two criteria were necessary for the successful transport of a nanoparticle to a target spot. One is the half-life of NPs in circulation, and the other is nanoparticle targeting. The size, charge, and coating materials of NPs have a considerable influence on the half-life of NPs. It has been observed that nanomedicines with a size of approximately 100 nm have a longer half-life in the bloodstream, but those with a size over 200 nm activate the lymphatic system and are more quickly removed from circulation [32–33]. The core size and hydrodynamic size of our nanoparticles were around 100 nm and 245 ± 3 nm, respectively, making them suitable for intravenous delivery. Thus, the single emulsion/evaporation approach employing 3% PVA as the surfactant was suitable for encapsulating iron oxide nanoparticles, CHL, and IR780, which created stable nanoparticles with the desired size. Additionally, it was documented that nanoparticles with a hydrodynamic size above 100 nm are incapable of traversing the endothelium and the glomerular basement membrane during glomerular filtration in the kidney [34]. Thus, it may be assumed that the half-life of our NPs in the bloodstream is longer than that of smaller NPs.

In addition to the size of nanoparticles, negatively charged NPs are more likely to have an extended half-life. When NPs are administered, they come into contact with blood cells and plasma proteins, which may cause adsorption or opsonization by serum proteins [35]. However, these proteins will have a reduced probability of interacting with our negatively charged nanoparticles, as most proteins are likewise negatively charged. Another element influencing NP elimination is glomerular filtration. However, endothelial cell surfaces are negatively charged [35], therefore less NPs will be excreted through the kidneys.

In regard to extending the half-life of NPs in the bloodstream, the stealth surface of NPs also helps to maintain its stability. Poly(ethylene glycol) (PEG) on the surface of NPs would serve as a brush to inhibit serum protein adsorption [4]. The PEO block of F127 shares the same core structure as PEG; hence, the emergence of a form of PEG would likewise improve the pharmacokinetics of our NPs.

Second, the ligand attached to the nanoparticles would aid in the accumulation of nanoparticles within the cells. Numerous studies have employed different ligands to target overexpressed receptors on cancer cells, including folate receptors [13,37], integrins [36–37], and vascular endothelial growth factor (VEGF) receptors [38–39]. The folate receptor was selected as a targeting modality in our investigation. The findings of the uptake assays (Figure 3), fluorescent assay (Supporting Information File 1, Figure S2) and the cytotoxicity testing (Figure 4) revealed that F127-folate@NP and F127@NP had the same impact. However, both NPs produced superior outcomes than those for PVA@NP control. It has been demonstrated that F127 can enhance the cellular uptake of NPs in vitro via clathrin-mediated endocytosis and caveolae-mediated endocytosis [31,40]. The absorption of F127 onto nanoparticle surfaces would assist the NPs to enter the cells. Therefore, F127 has been used as a nanoparticle component for drug delivery. For example, doxorubicin-loaded L61/F127 NPs (SP1049C, Supratek Pharma Inc., Montreal, Canada) have reached phase three in clinical trials [41–42].

The F127-folate@NP has not shown a substantial increase in cell uptake, probably because of the excess of folate moiety on the NP surface. Due to its hydrophobicity, the folate moiety may bind to itself, reducing the targeting impact. In a recent investigation of PLGA nanoparticles, we discovered that PLGA nanoparticles did not develop when 100% PLGA-PEG-folate was employed [22]. When the weight ratio of PLGA-PEG and PLGA-PEG-folate was decreased from 50 to 1, the nanoparticles exhibited significantly improved uptake capabilities. The combination of F127 and F127-folate may thus be investigated in our next work. Another explanation could be that the cell culture media in the study was not suitable for the targeting of NPs due to the presence of folic acid in it. Therefore, using folic-acid-free cell culture media would be the option for targeting evaluation.

The uptake of both F127-folate@NP and F127@NP into the cells has shown the potential application not only for cancer therapy but also for diagnosis. First, the NPs are internalized into the cells and gradually degrade in the low pH of the endosomes releasing CHL to interrupt the cell cycle. Second, the NPs carry both imaging agents, IR780 and IO, which are suitable for near-infrared imaging and MRI. IR780 is used as the tracer during operation and also in phototherapy [23–24]. The IO NPs have been used as a contrast agent in MRI for diagnosis. Since MRI is a noninvasive and nonradiation technique, it could be used multiple times to follow therapy stages.

## Conclusion

We have formulated F127@NP and F127-folate@NP nanoparticles coated with F127 and F127-folate, respectively, using the emulsion/evaporation technique. The synthesized NPs showed the appropriate size and charge for systematic applications. The NPs also showed the ability to carry therapeutic drugs and deliver them into cells. Besides, they also showed the potential to be used as imaging agents for tracing and diagnosis.

## Supporting Information

File 1 Supplementary data.

## Data Availability

All data that supports the findings of this study is available in the published article and/or the supporting information to this article.
