# Supplementary material for: Nanocarrier systems loaded with IR780, iron oxide nanoparticles and chlorambucil for cancer theragnostics
Source: Beilstein J Nanotechnol. 2024 Feb 6;15:180–9. doi: 10.3762/bjnano.15.17 (PMC10862130; doi:10.3762/bjnano.15.17)
Supplement: File 1 — Supplementary data. [file Beilstein_J_Nanotechnol-15-180-s001.pdf]

BEILSTEIN JOURNAL OF NANOTECHNOLOGY

Supporting Information

for

Nanocarrier systems loaded with IR780, iron oxide nanoparticles and chlorambucil for cancer theragnostics

Phuong-Thao Dang-Luong, Hong-Phuc Nguyen, Loc Le-Tuan, Xuan-Thang Cao, Vy Tran-Anh and Hieu Vu Quang

Beilstein J. Nanotechnol. **2024,** *15,* 180–189. doi:10.3762/bjnano.15.17

Supplementary data

License and Terms: This is a supporting information file under the terms of the Creative Commons Attribution License (https://creativecommons.org/licenses/by/4.0). Please note that the reuse, redistribution and reproduction in particular requires that the author(s) and source are credited and that individual graphics may be subject to special legal provisions.
The license is subject to the *Beilstein Journal of Nanotechnology* terms and conditions: (https://www.beilstein-journals.org/bjnano/terms)

Supplementary data 1

Synthesis of F127-folate

The synthesis of F127-folate was done as previously reported [1].

The structure of F127-folate was confirmed by FTIR (Agilent Cary 630), conducting scans from 650 to 4000 cm^{-1} at a resolution of 4 cm^{-1} (Figure S1). The peaks in the box from 1600 to 1700 cm^{-1} show the appearance of ester bonding between F127 and folic acid. The result agrees with a previous study [2].

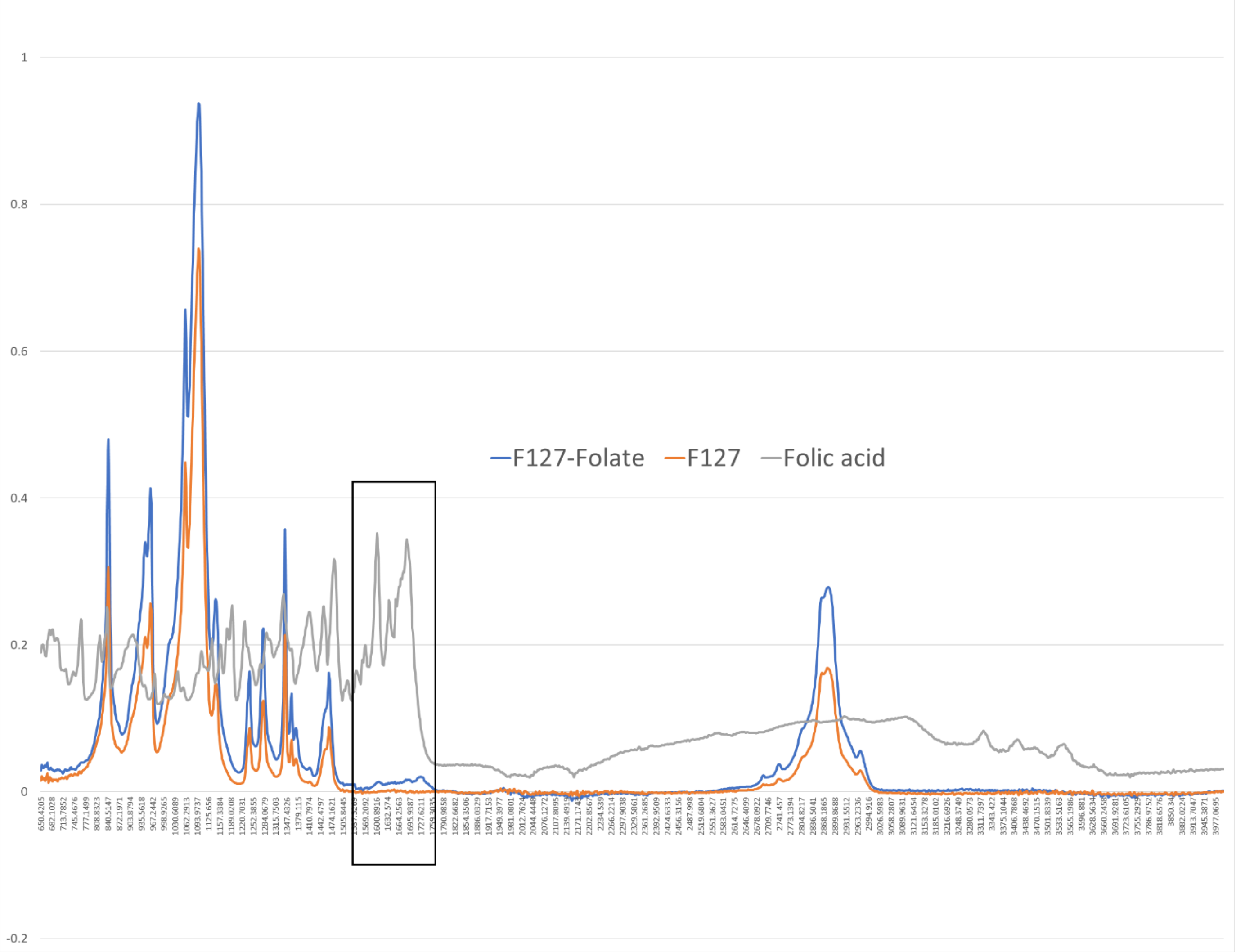

Figure S1: The FTIR of the F127-folate, F127 and folic acid. The square show the ester bonding of between F127 and folic acid from 1600 to 1750 cm^{-1}.

Supplementary data 2

Uptake of the NP to the cells

MCF7 and HepG2 were seeded onto 24-well plates overnight. The cells were incubated with 1 mg/mL of F127-Folate@NP/Cou-6/IO/IR780 and F127@NP/Cou-6/IO/IR780 for 3 h. Then the cells were washed with PBS three times. The fluorescence of the NPs in the cells was obtained using Nikon 80i Fluorescence microscope (Japan) (Figure S2). The result showed the signal from the NPs in both MCF7 and HepG2.

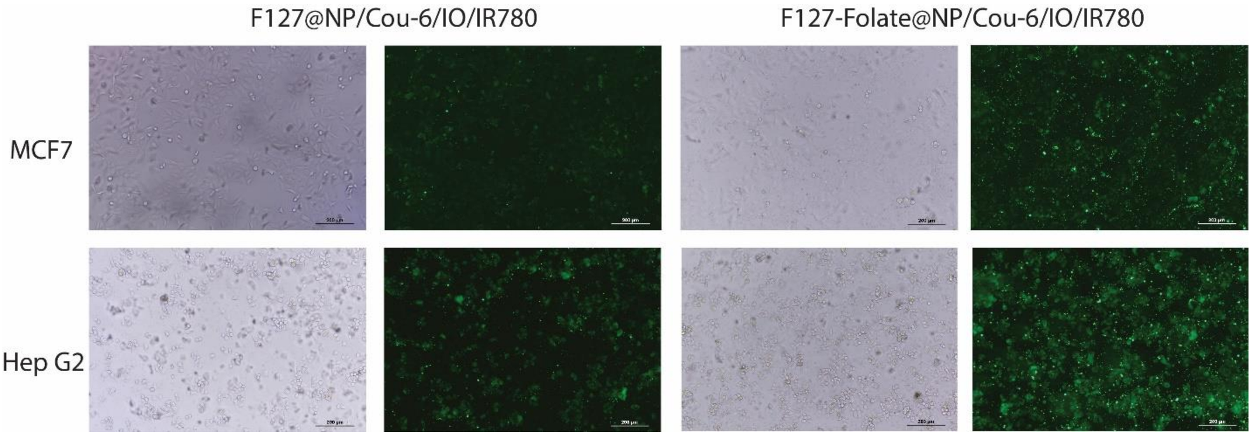

Figure S2: The fluorescence of F127@NP/Cou-6/IO/IR780 and F127-folate@NP/Cou-6/IO/IR780 to MCF7 and HepG2.

References

(1) Vu-Quang, H.; Vinding, M. S.; Nielsen, T.; Ullisch, M. G.; Nielsen, N. C.; Nguyen, D.-T.; Kjems, J. Pluronic F127-Folate Coated Super Paramagenic Iron Oxide Nanoparticles as Contrast Agent for Cancer Diagnosis in Magnetic Resonance Imaging. *Polymers (Basel)* **2019**, *11* (4). https://doi.org/10.3390/polym11040743.

(2) Costa, K. M. N.; Barros, R. M.; Jorge, E. O.; Sato, M. R.; Chorilli, M.; de Lima Damasceno, B. P. G.; Nicholas, D.; Callan, J. F.; Oshiro Junior, J. A. Doxorubicin-Loaded Nanostructured Lipid Carriers Functionalized with Folic Acid against MCF-7 Breast Cancer Cell Line. *Journal of Nanoparticle Research* **2023**, *25* (4), 56. https://doi.org/10.1007/s11051-023-05704-7.
